# Comparison of Prophylactic Infusion of Phenylephrine Versus Norepinephrine for the Prevention of Post Spinal Hypotension in Parturients Undergoing Elective Caesarean Section-a Randomized, Double-Blinded, Non-Inferiority Trial

**DOI:** 10.4274/TJAR.2022.22909

**Published:** 2023-06-16

**Authors:** Banupriya Ravichandrane, Rajeshwari Subramaniam, Thilaka Muthiah, Praveen Talawar, Rajasekar Ramadurai

**Affiliations:** 1 Department of Anaesthesiology, All India Institute of Medical Sciences, Pain Medicine and Critical Care, New Delhi, India; 2 Department of Anaesthesiology, All India Institute of Medical Sciences, Rishikesh, India; 3 Department of Anaesthesiology & Critical Care, Jawaharlal Institute of Postgraduate Medical Education and Research, Pondicherry, India

**Keywords:** Cesarean section, norepinephrine, obstetric anaesthesia, phenylephrine, postspinal hypotension

## Abstract

**Objective:**

Postspinal hypotension occurs in nearly 50% of women undergoing cesarean section (CS). Although phenylephrine (PE) is currently the vasopressor of choice, severe maternal bradycardia may adversely affect the fetal status due to the reduction in the maternal cardiac output. Norepinephrine (NE) is not associated with bradycardia and is now being evaluated for the treatment of post-spinal hypotension in obstetric patients. The hypothesis of this study was that the prophylactic NE infusion was non-inferior to PE infusion when used for the prevention of postspinal hypotension.

**Methods:**

This was a randomized, double-blinded controlled study conducted in 130 parturients scheduled for CS. The participants received either prophylactic NE (5 μg min^-1^) or PE (25 μg min^-1^) infusion beginning at the time of spinal injection. The primary outcome was the incidence of hypotension in both groups. Maternal bradycardia, reactive hypertension, nausea and vomiting, requirement of rescue boluses of vasopressor and/or atropine, and neonatal acid base status were also recorded.

**Results:**

The incidence of hypotension was 33.80% (22 of 65) in Group PE and 26.10% (17 of 65) in Group NE (*P*=0.85). The absolute risk difference [90% confidence interval (CI)] in the incidence of hypotension between the groups was -7.7% (-20.9, 5.4). The upper limit of the CI was less than the non-inferiority margin of 20%, indicating that the NE infusion was non-inferior to PE.

**Conclusion:**

Prophylactic infusion of NE is not inferior to prophylactic PE infusion in the prevention of postspinal hypotension in patients undergoing CS.

**Main Points**

• Hypotension is one of the most common consequences of spinal anaesthesia.

• Vasopressors like phenylephrine are the primary agents used for the management of post spinal hypotension.

• Phenylephrine-induced reflex bradycardia can be deleterious to the fetus.

• Norepinephrine was found to be equally effective in treating post spinal hypotension.

## Introduction

Spinal anaesthesia is commonly preferred over general anaesthesia in parturients undergoing elective cesarean section (CS). Hypotension occurs in nearly 80% of the parturients due to the blockade of preganglionic sympathetic neurons and subsequent fall in systemic vascular resistance.^1^

Phenylephrine (PE), a pure alpha (α) agonist, has emerged as the vasopressor of choice for the management of post spinal hypotension since it has less propensity to depress fetal pH and base excess than ephedrine.^2,3^ The associated bradycardia has a theoretical potential of causing a fall in maternal cardiac output and subsequent impact on the fetus. Hence the usage of norepinephrine (NE), an α1 adrenergic agonist with weak beta (β)1 adrenergic agonist activity, with minimal changes in maternal HR has been suggested recently.^4,5^

This randomized, prospective, double-blinded, controlled trial was designed to compare the efficacy of prophylactic intravenous (IV) infusion of PE and NE in the prevention of post spinal hypotension in parturients undergoing elective CS. The primary outcome measure was the incidence of hypotension between the groups. We hypothesized that NE is equally effective or not inferior to PE in the management of postspinal hypotension for elective CS. Secondary outcome measures included incidence of maternal bradycardia, nausea and vomiting, reactive hypertension, requirement of rescue boluses of vasopressor and/or atropine, and neonatal acid base status.

## Methods

This prospective, randomized controlled double-blinded study was conducted after obtaining approval from the institute ethics committee. Written informed consent was obtained from all participants. The study was registered with Clinical Trials Registry of India at clinicaltrials.gov. The study was conducted over a period of 14 months (from August 2017 to October 2018). The manuscript has been prepared in accordance with the revised 2010 CONSORT guidelines, incorporating extra points from “Extension of CONSORT 2010 checklist when reporting a non-inferiority randomized trial”.^6^

The American Society of Anesthesiologists grade II parturients with singleton term pregnancy scheduled for elective CS were included in the study. Patients with severe systemic illness (uncontrolled diabetes, hypertension, cardiac disease etc.), obstetric complications (pregnancy induced hypertension, abnormal placentation), and patients in active labor were excluded from the study.

Randomization was achieved using a computer-generated random sequence. Patient codes along with instructions to prepare the drug were placed into sequentially numbered sealed opaque envelopes. A resident anaesthesiologist who was not involved in patient management prepared the drugs. The patient and the attending anaesthesiologist conducting the CS were blinded to the study drug.

**Anaesthesia Protocol:** Patients were fasted overnight and were given IV metoclopramide 10 mg and ranitidine 50 mg. On arrival to the operating room (OR), electrocardiogram, non-invasive blood pressure (BP), and SpO_2_ monitors were attached. Baseline heart rate (HR) and BP were noted by taking an average of three values recorded at an interval of 2 min in the OR with the patient in supine position with left lateral tilt. An 18G IV cannula was placed and coloading was achieved using 500 mL of lactated Ringer solution. Another wide-bore cannula was placed in the contralateral arm. Fetal HR was monitored by external cardicotocography until the commencement of surgery.

Subarachnoid block (SAB) was administered by an experienced anaesthesiologist (not necessarily the same person always) with 10 mg heavy bupivacaine and 150 µg of preservative free morphine in the L3-L4 interspace using a 25 G Quincke needle with the patient in the sitting position. After the block, patients were made supine with left lateral tilt, and vasopressor infusion was started at 15 mL h according to the group allocation:

Group 1 (later decoded as PE) patients received 25 µg min^-1^ of PE (diluted to reach a concentration of 100 µg mL).

Group 2 (later decoded as NE) patients received 5 µg min^-1^ of NE (diluted to reach a concentration of 20 µg mL).

There were no failed spinal blocks in either of the two randomized groups. The SAB was assessed until the loss of sensation for cold at the level of the T4-T5 dermatome and surgery was allowed to start. BP and HR values were recorded at intervals of every minute till the delivery of the baby and every 5 min till the end of surgery. Hypotension was defined as a decrease in systolic blood pressure (SBP) of >20% from baseline or the absolute value of SBP <100 mm of Hg, and was treated with a rescue bolus of PE 25 µg IV and repeated once more if there was no improvement in the SBP. In case of reactive hypertension, defined as an increase in SBP >20% from baseline, the study drug infusion was stopped. Patients with bradycardia (HR <50 beats min^-1^) were treated with atropine (0.3 mg IV bolus) and repeated if necessary.

The oxytocin infusion (10 IU in 500 mL normal saline) was started for all parturients after the delivery of the neonate. The study drug was continued till uterine closure and the data was recorded at the end of surgery. Paracetamol 1 g IV and ondansetron 4 mg IV were administered before the transfer of the patient to the postoperative recovery area.

**Data Collection:** The primary outcome of our study was to compare the incidence of maternal hypotension after SAB. Secondary outcomes were to compare the incidence of maternal bradycardia, reactive hypertension, nausea, and vomiting, requirement of rescue boluses of vasopressor and/or atropine, and neonatal acid base status (umbilical cord blood gases). Apgar scores were also noted in 1 min and 5 min post delivery.

### Statistical Analysis

The incidence of hypotension was reported as 30% in a previous study using the same dose of prophylactic PE infusion (25 µg min^-1^).^7^ For calculating a 90% confidence interval (CI) with a non-inferiority margin of 20%, a sample size of 65 patients was required per group, assuming a power of 80% and an alpha error of 0.05.

Statistical analysis was performed using Stata 12.0 (College Station, Texas, USA). Data were presented as mean ± standard deviations or number (percentage) or median (range) as appropriate. Continuous baseline characteristics were compared using an unpaired *t*-test (area under the curve for episodes of hypotension and neonatal APGAR scores and umbilical blood gas parameters) or Wilcoxon-rank-sum test (spinal induction to incision time and uterine incision to delivery time). The categorical variables were compared using the chi-square test or Fisher’s exact test (parity, incidence of hypotension/bradycardia and requirement of rescue boluses) as appropriate. A *P* value of <0.05 was considered statistically significant. For the primary endpoint, the non-inferiority of prophylactic infusion of NE to PE was planned to be claimed if the difference (90% CI) in incidence of hypotension was less than the margin of 20%.

## Results

**Figure 1:**
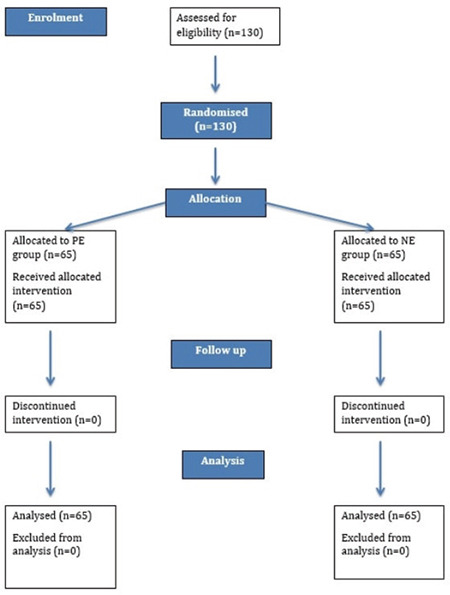
Consort flow diagram of the study.

One hundred and thirty patients consented and were randomly allocated to receive NE at 5 µg min (n = 65) or PE at 25 µg min (n = 65). All patients received the intended intervention and were available for final analysis (Figure 1). Patient demographics, as described in Table 1, were statistically non-significant between the groups.

**Figure 2:**
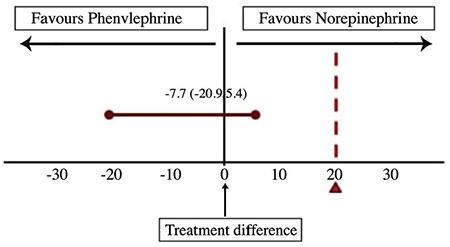
Figure showing the treatment difference between the groups. The upper limit of the 90% CI is less than the non-inferiority margin of 20%, which shows that prophylactic infusion of norepinephrine is non-inferior to the prophylactic infusion of phenylephrine in preventing post-spinal hypotension. CI, confidence interval.

Maternal outcomes are shown in Table 2. The difference in the incidence of hypotension with PE (22 out of 65, 33.8%) and NE (17 out of 65, 26.1%) was statistically non-significant [risk ratio: 1.29 (0.72, 2.29) *P* value-0.85. The difference (90% CI) in the incidence of hypotension between the groups was -7.7% (-20.9, 5.4) (Figure 2), which denote that NE was non-inferior to PE in preventing hypotension. The number of boluses of rescue vasopressor (*P*=0.48), pre-delivery HR (*P*=0.26), and post- delivery HR (*P*=0.74) were statistically non-significant between the groups.

The incidence of maternal bradycardia (HR <50 min) was 12.3% (8 out of 65) with PE and 10.7% (7 out of 65) with NE, which was statistically non-significant (*P*=0.46). The incidence of nausea was 9.2% (6 out of 65) with PE and 4.6% (3 out of 65) with NE, statistically non-significant (*P*=0.49) and was associated with hypotension in all patients; however, no patient had vomiting. Ventricular premature contractions (VPCs) occurred in 7 patients in the NE group as opposed to none in the PE group. Only 3 patients among 7 who had VPCs experienced reactive hypertension that required termination of infusion. None of these patients required further treatment.

The neonatal outcome was comparable between the groups (Table 3). The subgroup analysis in neonates born of patients with bradycardia in the PE group (n = 7) had lower umbilical cord pH compared to their counterparts in the NE group (n = 8), which approached significance (*P*=0.052).

## Discussion

The results of our study show that prophylactic infusion of NE is non-inferior to PE in maintaining maternal SBP after spinal anaesthesia. There was no statistical difference in the incidence of hypotension, maternal bradycardia, rescue bolus requirement of vasopressor, and neonatal effects between the groups. Post-spinal hypotension is common in parturients undergoing CS, with a decrease in systemic vascular resistance recognized as a significant contributor. Prophylactic administration of PE has been observed to be more effective than ephedrine in reducing the incidence of post spinal hypotension.^3^ PE infusions at higher rates (75 µg min^-1^ and 100 µg min^-1^) were associated with higher incidence of hypertension and bradycardia as compared to lower infusion rates (25 µg min^-1^ and 50 µg min^-1^).^7,8^ Hence we used the lowest effective dose (25 µg min^-1^) of prophylactic PE infusion to maintain the SBP in our study.

Studies using bolus PE have reported significant maternal bradycardia compared to NE infusion or NE infusion and ephedrine boluses.^9,10^ The reflex bradycardia associated with PE warranted the search for a new vasopressor; when NE (strong α-adrenergic with mild β-adrenergic action) was suggested as a reliable vasopressor for the management of post spinal hypotension.^4,11^ Different NE dosing regimens have been evaluated for prevention of post spinal hypotension in the obstetric setting. Chen et al.^12^ observed that NE at 5 and 10 µg kg^-1^ h^-1^ maintained BP with less episodes of reactive hypertension compared to 15 µg kg^-1^ h^-1^. Since the relative potencies of NE and PE compared in previous studies ranged from 20:1 to 2:1, with no defined optimal potency ratio; we used infusions of NE at 5 µg min^-1^ and PE at 25 µg min^-1^, the doses associated with minimal adverse effects.^5,11,13^

**Figure 3:**
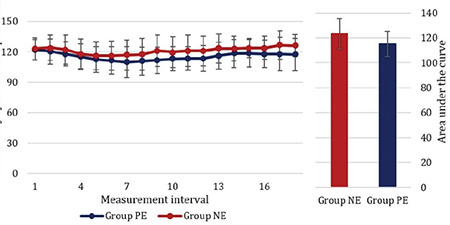
Figure showing trends of maternal pre-delivery systolic blood pressure. PE, phenylephrine; NE, norepinephrine.

The incidence of hypotension observed in our study (33.8% with PE vs. 26.1% with NE) was similar between the groups and comparable to the previous works done with equivalent doses of vasopressors.^7,8,14^ The predelivery SBP over time in the present study was significantly higher in the NE group (123.4 ± 12.2 mmHg) compared to the PE group (115.3 ± 10.2 mmHg) (*P*=0.04) (Figure 3) and the difference (90% CI) in incidence of hypotension between the groups was observed to be less than the margin of 20%, inferring that prophylactic infusion of NE is non-inferior (as effective as) to the prophylactic infusion of PE in preventing post-spinal hypotension. The requirement for rescue vasopressor was also similar between the groups. These findings are in agreement to the findings of Ngan et al.^5^ and Vallejo et al.^11^

In the present study, the observed incidence of bradycardia (12.3% with PE and 10.7% with NE) was comparable with Allen et al.'s^7^ (15% with 25 µg min^-1^ of PE) and Vallejo et al.'s^11^ studies (23.7% with PE and 18.6% with NE). The reported higher incidence of bradycardia with PE compared to NE in previous studies could be due to the usage of relatively higher dose of PE compared to NE.^5^ However, in this study, there was no significant difference in the incidence of bradycardia between the groups, likely due to the use of the lowest effective dosage. Chen et al.^15^ also reported no significant difference in the incidence of bradycardia between prophylactic NE (3.2  µg min^-1^) and PE (40  µg min^-1^) infusion in twin pregnancy. Though the clinical importance of PE induced bradycardia remains uncertain in elective CS, it might have some possible adverse impact in the presence of pre-existing fetal compromise.^16^

There was no significant difference in neonatal Apgar scores and umbilical artery pH between the groups. However, a subgroup analysis of the umbilical artery pH of neonates born to mothers who developed bradycardia revealed that the PE group were more acidotic (7.26 ± 0.03) than the NE group (7.29 ± 0.06), *P*=0.05. This is in concordance with Ngan et al.^5^, who also reported significantly lower umbilical venous pH in neonates born of mothers receiving PE. This subgroup analysis cannot be generalized since only a few mothers had bradycardia in our study. However, it is worth considering that bradycardia in mothers receiving PE could be a marker for reduced CO despite “normal” BP, which may further affect a compromised fetus. Similar to our findings, Ngan et al.^17^ in his recent study, reported that NE was non-inferior to PE for neonatal outcome assessed by umbilical arterial pH.

The incidence of nausea and vomiting was found to be similar in both groups in this study. There was also a positive correlation between hypotension and nausea, which could be due to cerebral hypoperfusion. The incidence of reactive hypertension with NE infusion is a dose-dependent effect. The episodes of reactive hypertension with NE infusion, required cessation of infusion.^11,12^ In this study, only 3 of 65 patients (4.6%) in the NE group had reactive hypertension, which is lower than the reported literature. In spite of the apparently “normal” dosing of NE in our study, seven patients had episodes of ventricular ectopics that resolved spontaneously. This could be directly attributed to NE because three of these patients also had a hypertension, necessitating the cessation of NE infusion.

The limitations of this study are as follows. Invasive BP measurement was not chosen for ethical reasons although the accuracy of BP measurements would have been enhanced. Administration of PE rescue bolus in both groups might have also affected the hemodynamics and could have biased the results. CO monitoring may have been more informative in this setting but was not used in our study due to non-availability. A control group could have widened the comparability and possibly explained the higher incidence of hypotension despite preloading and use of prophylactic inotropic infusions in both groups.

## Conclusions

Prophylactic infusion of NE (5 µg min^-1^) was observed to be equally effective (non-inferior) in the prevention of post-spinal hypotension in patients undergoing elective CS compared with PE infusion (25 µg min^-1^). The neonatal effect of doses of PE resulting in maternal bradycardia must be further evaluated stringently. In case the significant fetal acidosis does occur, NE may emerge as the vasopressor of choice for the prevention and treatment of post-spinal hypotension in obstetrics.

## Figures and Tables

**Table 1:**
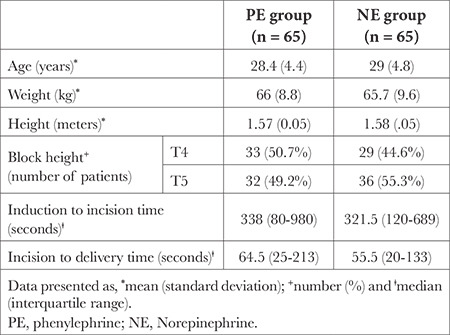
Patient Demographics and Operative Data

**Table 2:**
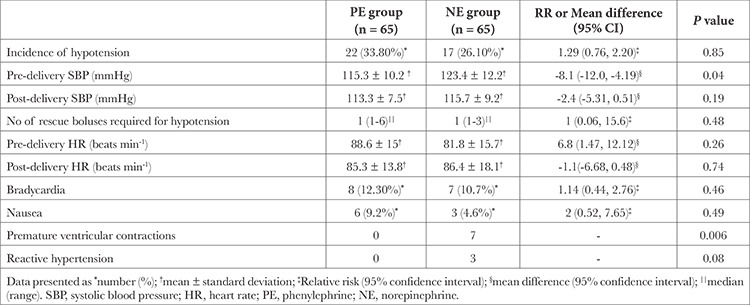
Maternal Outcomes

**Table 3:**
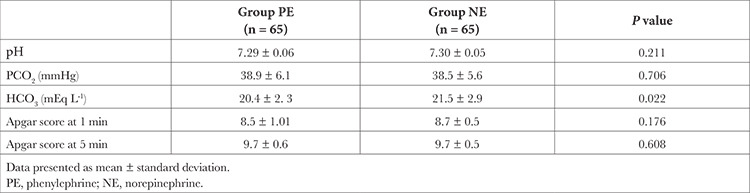
Neonatal Parameters
